# How Much Can We do by Reverse Superficial Sural Artery Flap to Potentiate Its Effects: Introducing Spout Sural Flap as a New Modification

**Published:** 2017-09

**Authors:** Jalaluddin Khoshnevis, Terifeh Dashti, Eznollah Azargashb, Mohamad Reza Kalantar Motamedi

**Affiliations:** 1 Department of General and Vascular Surgery, Shohada-e-Tajrish Medical Center, Shahid Beheshti University of Medical Sciences, Tehran, Iran;; 2 Department of Health Service Management, Clinical Research Development Center of Shohadaye Tajrish Hospital, Shahid Beheshti University of Medical Sciences, Tehran, Iran;; 3 Department of Community Medicine, Shahid Beheshti University of Medical Sciences and Health Services, Shohadaye Tajrish Hospital, Tehran, Iran

**Keywords:** Reverse, Superficial, Sural artery flap, Foot, Spout, Delayed

## Abstract

**BACKGROUND:**

Due to shortage of local donor tissue and unreliable blood supply, free flaps were the mainstay of treatment for tissue defects in the lower leg and foot region, but it requires a qualified microvascular surgeon. Recently, attention has been paid to reverse superficial sural artery flap (RSSAF) and its modifications as a good alternative to pave the way to simple and friendly techniques.

**METHODS:**

Excluding each patient with septic and severely ischemic foot, every patient with tissue defect in distal leg and proximal foot region were studied. Various methods were applied including spout technique with sufficient follow up. No imaging was used to evaluate the blood supply.

**RESULTS:**

Five patients underwent spout technique with excellent results in four cases. Spout technique in one case failed due to narrow base. In five cases, RSSAF was performed with creating skin tunnel and very good results.

**CONCLUSION:**

RSSAF is a good alternative for free flap to cover the leg and foot tissue defects. We also advise wide base pedicle (>4 cm) in every patient.

## INTRODUCTION

Due to unreliable blood supply and paucity of local donor tissue, free flaps have become the mainstay of treatment for the traumatized lower limb over the past decades.^1^^,^^2^ Its drawbacks include longer operative time, potential donor site morbidity and the need for qualified surgeons with microsurgical experience.^3^ The reverse superficial sural artery flap (RSSAF) is a reverse based fasciocutaneous or adipofascial flap that is increasingly being used for coverage of defects that involve the distal third of the leg, ankle, and foot. Significant advantages of this flap which are constant blood supply without sacrifice or manipulation of a major artery to the lower limb.^4^^-^^6^ Recent meta-analysis showed that 82% of flaps heal without any flap-related complications.^7^

The RSSAF is often at risk for venous congestion, as it relies on communication between the venacomittants of the sural nerve and the lesser saphenous vein, thus circumventing the valves of the deep venous system.^8^ Impaired venous drainage is an important cause of failure in the early postoperative period.^7^^-^^9^ It has been shown that flap survival was improved by various modifications to the operative technique that enhanced venous outflow and that these changes reduced the use of leech therapy.^7^^,^^10^^,^^11^ We presented our experience with spout pedicle which not only affected on wider base, but also on prevention of kinking of pedicle and pressure on it. 

## MATERIALS AND METHODS

Patients who had septic wound or their heel bone was exposed and ischemic were excluded from the study. So four patients were excluded for septic wounds and one patient for ischemic heel bone. Finally, 10 patients were included for study. One of them was included after the sepsis of foot that was controlled (Case 2). Our cases were various and we planned sural flap according to their variety. We compared our findings with recent meta-analysis, considering similarities and differences.

## RESULTS

Ten cases, while nine of them were male and one was female were 20-70 years old (mean=45 years). Ultimately RSSAF was undertaken. The included patients were two subjects with diabetic chronic ulcers, two with malignant lesions, five with traumatic chronic wounds, and one with unknown etiology (Table 1). We performed five island flap with wide base (4 cm) whose pedicle was adipofascial type and passed through the skin tunnel with excellent healing. Five patients underwent spout sural flap (Defined when the pedicle passed over the skin not through the skin tunnel), whie two cases were in delayed manner and three cases in immediate manner. Three of spout pedicles were completely bare without any coverage even with split thickness skin graft. One pedicle had coverage partially with its own skin over and one of them was surrounded completely by its overlying skin. 

**Table 1:** Demography of included patients

**No**	**Age (y)**	**Sex**	**Etiology of the wound**	**Type of flap and pedicle**	**Size (cm** ^2^ **)**	**Pedicle base width (cm)**	**Follow up (y)**	**Results**
1	70	Male	Diabetic+ Marjolin [burn]	Delayed, fasciocutaneous pedicle coverd partially with skin [spout]	36	4	8	Excellent
2	63	Male	Diabetic wound	Delayed, fasciocutaneous pedicle which completely coverd by skin [spout]	48	4	10	Excellent
3	30	Male	Squamous cell carcinoma	Island Flap, adipofascial pedicle [spout]	9	4	2	Excellent
4	55	Male	Melanoma	Island flap, adipofascial pedicle, passed through the skin tunnel	16	4	1	Excellent
5	45	Male	Trauma, heel bone fistula	Island flap, adipofascial pedicle, passed through the skin tunnel	20	4	4	Excellent
6	55	Female	Melanoma	Island flap, adipofascial pedicle, spout	100	4	5	Excellent
7	25	Male	Trauma, chronic wound	Island flap, adipofascial pedicle, passed through the skin tunnel	25	4	3	Excellent
8	20	Male	Trauma, chronic wound	Island flap, adipofascial pedicle, passed through the skin tunnel	20	4	2	Excellent
9	35	Male	Trauma, Chronic wound	Island flap, adipofascial pedicle, passed through the skin tunnel	12	4	1	Excellent
10	57	Male	Chronic wound, unknown etiology	Island flap, bare adipofascial pedicle [spout]	49	2	1 month	Failure

**Fig. 1:**
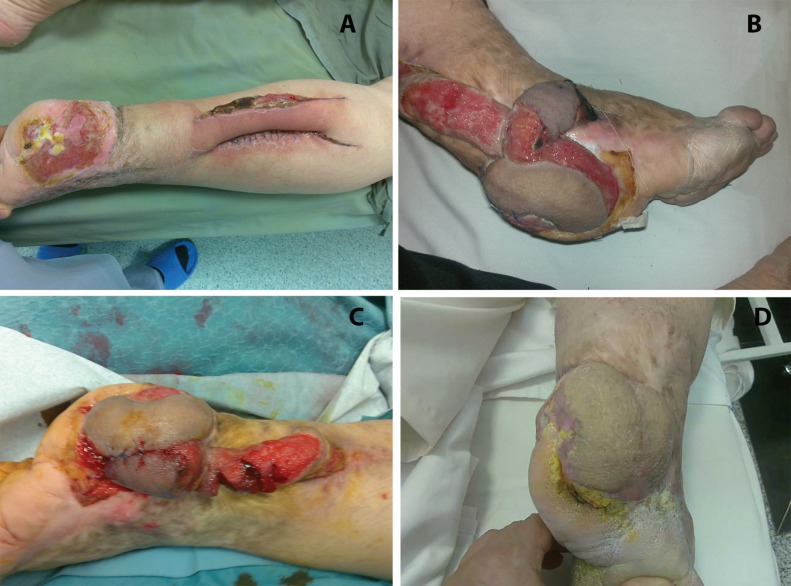
Case 1. Spout RSSAF, partially bare (a) Diabetic marjolin heel wound with delayed spout partially covered pedicle (b) Partially covered spout flap. Reversed rotation flap created at second operation (c) Partially covered spout flap. Its skin was used as a reversed flap (d) Final result

**Fig. 2:**
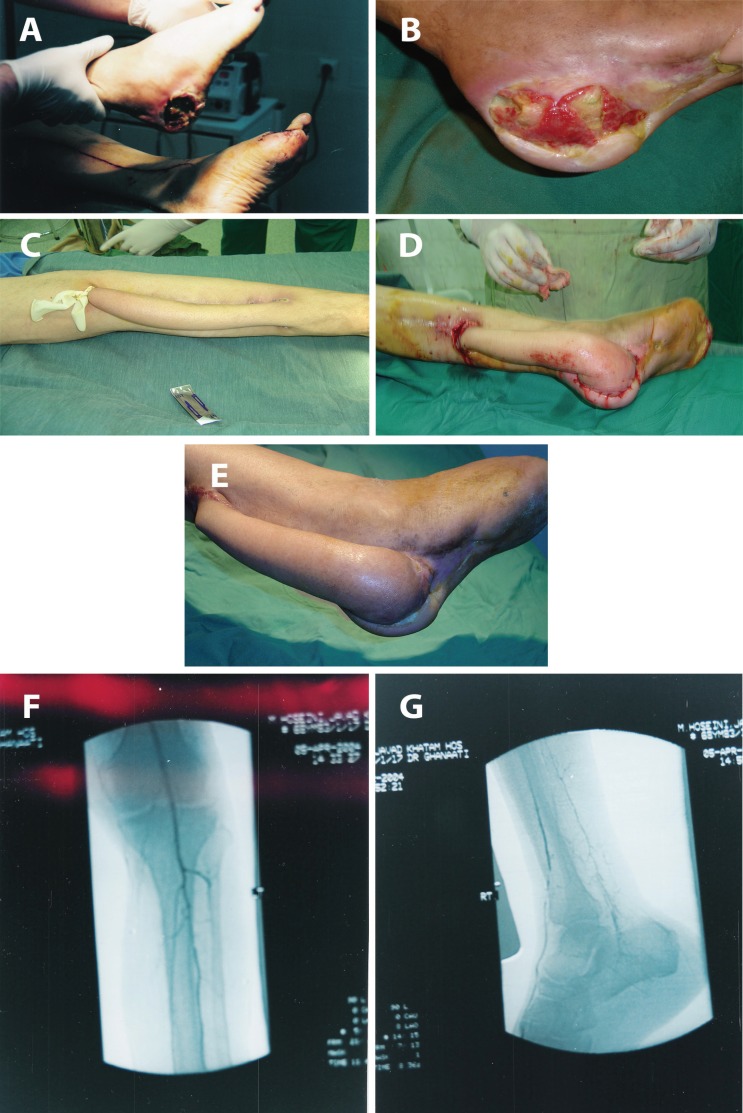
Case 2. Spout RSSAF, completely covered by skin (a) diabetic wound after debridment (b) Wound after prolonged management. See granulation tissue over bone (c) Design of delayed spout sural flap (d) Spout delayed sural flap with complete coverage of pedicle (e) Spout RSSAF after healing (f) Angiography of this case (g) Angiography lateral view

**Fig. 3:**
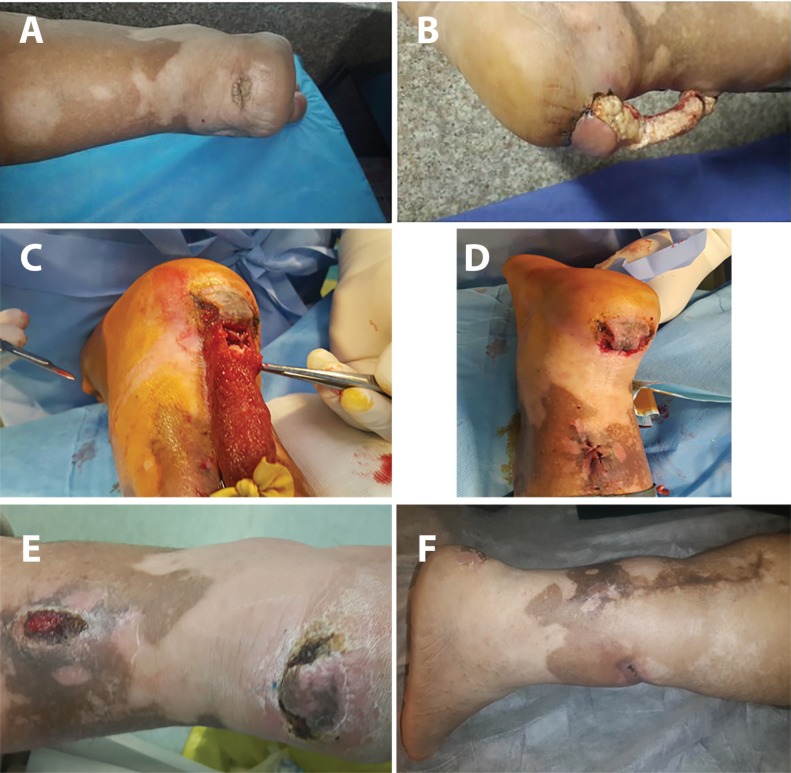
Case 3. Spout RSSAF, completely bare (a) Squamous cell carcinoma of heel (b) SSAF. Spout pedicle without skin graft (c) Spout RSSAF, 3 weeks later with growth of granulation tissue over pedicle (d) Spout RSSAF, after pedicle removal 3 weeks later (e) Spout RSSAF, 6 weeks after pedicle removal (f) Spout RSSAF, 1 year after pedicle removal

**Fig. 4:**
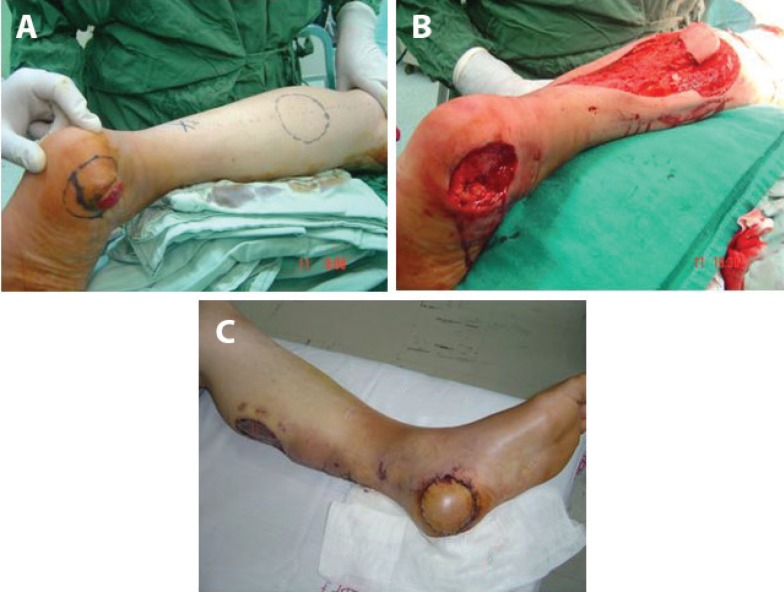
Case 4. RSSAF. (a) Melanoma and design of RSSAF (b) Excision of the lesion and performance of RSSAF through the skin tunnel (c) RSSAF 1 week postoperatively

**Fig. 5:**
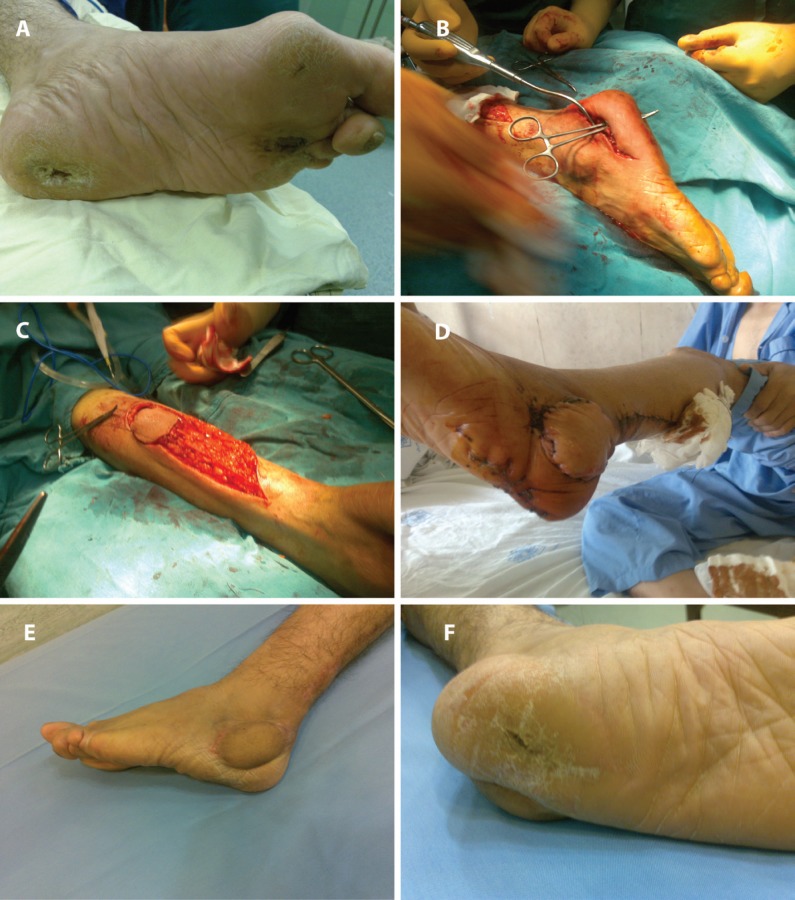
Case 5. RSSAF (a) CRF, candidate for renal transplantation,note calcaneal fistula due to trauma (b) Calcaneal bone fistula due to trauma (c) RSSAF design (d) Pedicle through the skin tunnel (e) lateral wound coverage with pedicle passed through skin tunnel and see healing of lateral hole of fistula (f) Healing of fistula and the case is ready for renal tranplantation

**Fig. 6:**
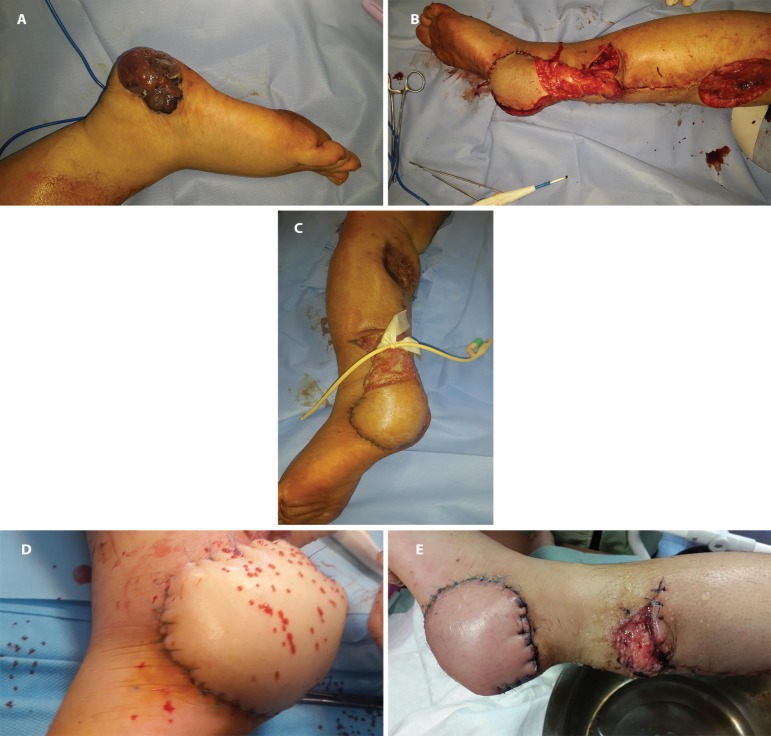
Case 6. Spout RSSAF, completely bare (a) Malignant Melanoma (b) Spout RSSA flap with bare pedicle (c) Spout RSSA flap with bare pedicle .Ligation after 3 weeks (d) Spout RSSA flap,After pedicle removal (e) Spout RSSAF, one week after pedicle removal

**Fig. 7:**
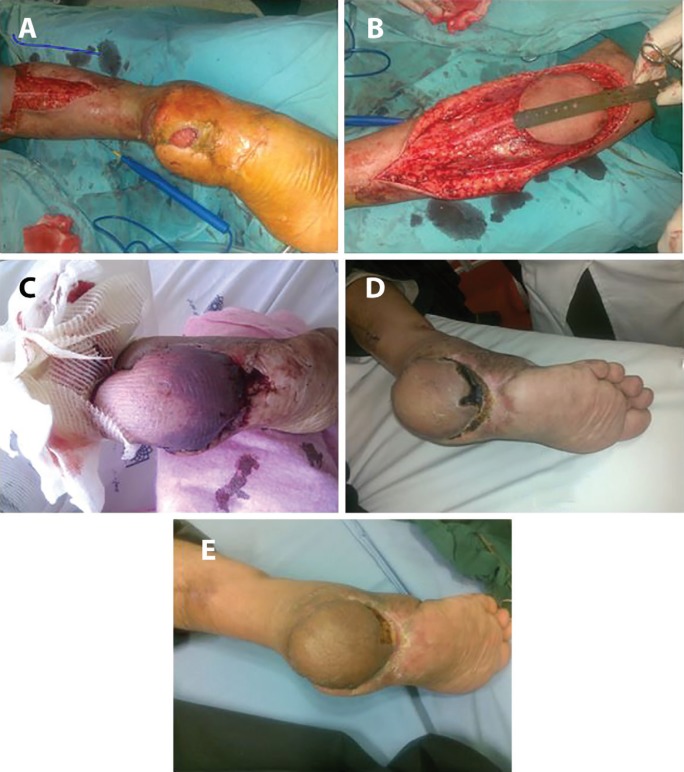
Case 7. Spout RSSAF, Traumatic,bare (a) Traumatic heel wound. (b) Design of RSSAF (c) Spout Flap of RSSAF without skin graft of pedicle (d) Spout RSSAF after pedicle removal. See minimal loss (e) Spout RSSAF ,after removal of pedicle,late result

**Fig. 8:**
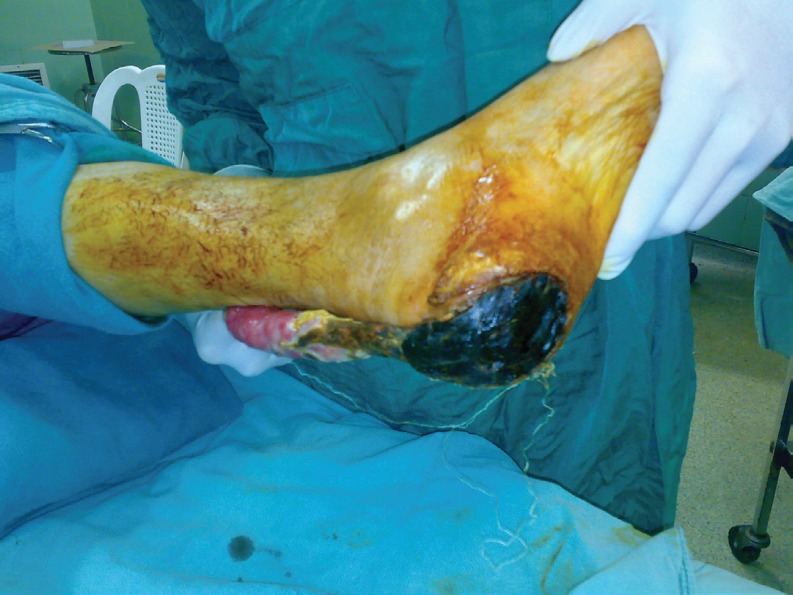
Spout RSSAF. Failure due to narrow base and bad cooperation

Healing was excellent in nine cases and very bad in one case. RSSAF was successful as a delayed flap, especially in patients which usually did not have good vessel for free flaps (Figure 1 and 2). We covered its pedicle partially or completely with its own skin (Figure 1 and 2) or let it to be bare (Figure 3-7). The flap was extended to popliteal fossa in both immediate and delayed manner (Figures 2, 5 and 7). In lean patients, the adipofacial pedicle was passed through the skin tunnel (Figure 4 and 5). The only failure was in the spout pedicle group with the heel wound of unknown etiology, the pedicle base of narrow (2 cm) and the patient of uncooperative (Table 1: Case 10, Figure 8). 

## DISCUSSION

RSSAF has been recognized as a good alternative to free flaps.^1^ Further effort for technique revision will result in better outcome. According to new meta-analysis we also find that taking a wide base flap can prevent kinking and venous congestion.^7^^-^^10^ We also experienced sural flap, successful as a delayed flap, especially in patients which usually have not good vessel for free flaps. We extended the flap to popliteal fossa in both immediate and delayed manner. Also we covered its pedicle partially or completely with its own skin or let it to be bare. In lean patients we passed the adipofacial pedicle through the skin tunnel. This technique in fatty cases may lead to kinking, ischemia, pressure and gangrene of the flap. Five patients underwent RSSAF with pedicle as spout sural flap. Considering a spout flap with bare pedicle which allows to close the pedicle harvesting site primarily without more dissection and without any consequences makes it user friendly and patient satisfaction.^7^^-^^10^

One of our spout sural flap which failed was in an uncooperative patient with an ulcer of unknown etiology under the heel and narrow base. The failure was most probable due to pressure in the middle of pedicle and kinking due to narrow base. We showed the impact on the pedicle base width of at least 4 cm. Additionally, we introduced spout pedicle especially in fatty or diabetic patients with its variations including delayed sural flap, conventional superficial sural artery flap with or without skin coverage of pedicle as a successful technique.
